# Supplementary material for: Soil depth determines the microbial communities in Sorghum bicolor fields within a uniform regional environment
Source: Microbiol Spectr. 2025 Apr 16;13(6):e02928-24. doi: 10.1128/spectrum.02928-24 (PMC12131775; doi:10.1128/spectrum.02928-24)
Supplement: Supplemental figures — Fig. S1 to S3. [file spectrum.02928-24-s0001.pdf]

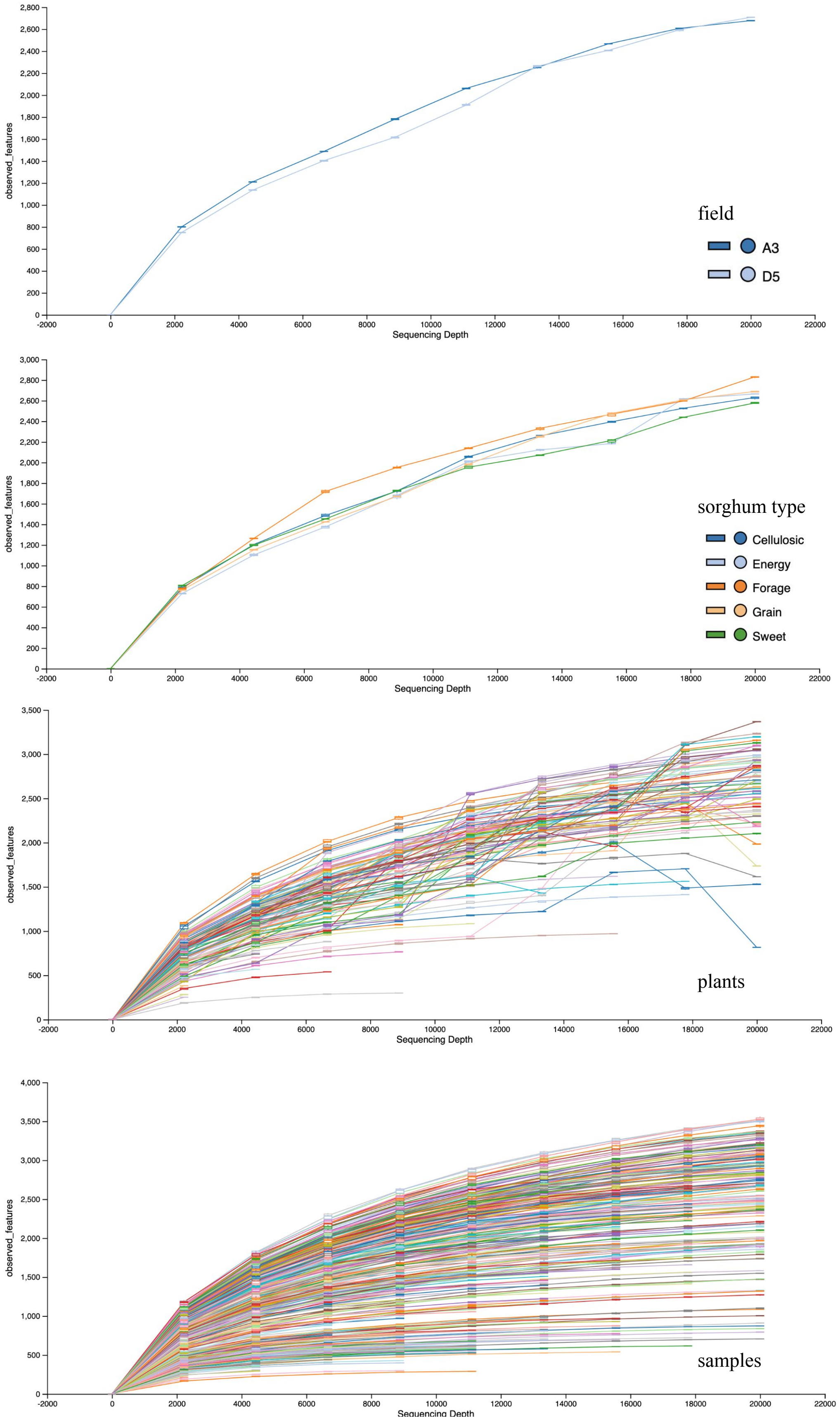

Fig S1:Rarefaction curves showing observed features across fields, sorghum types, individual plants, and samples.

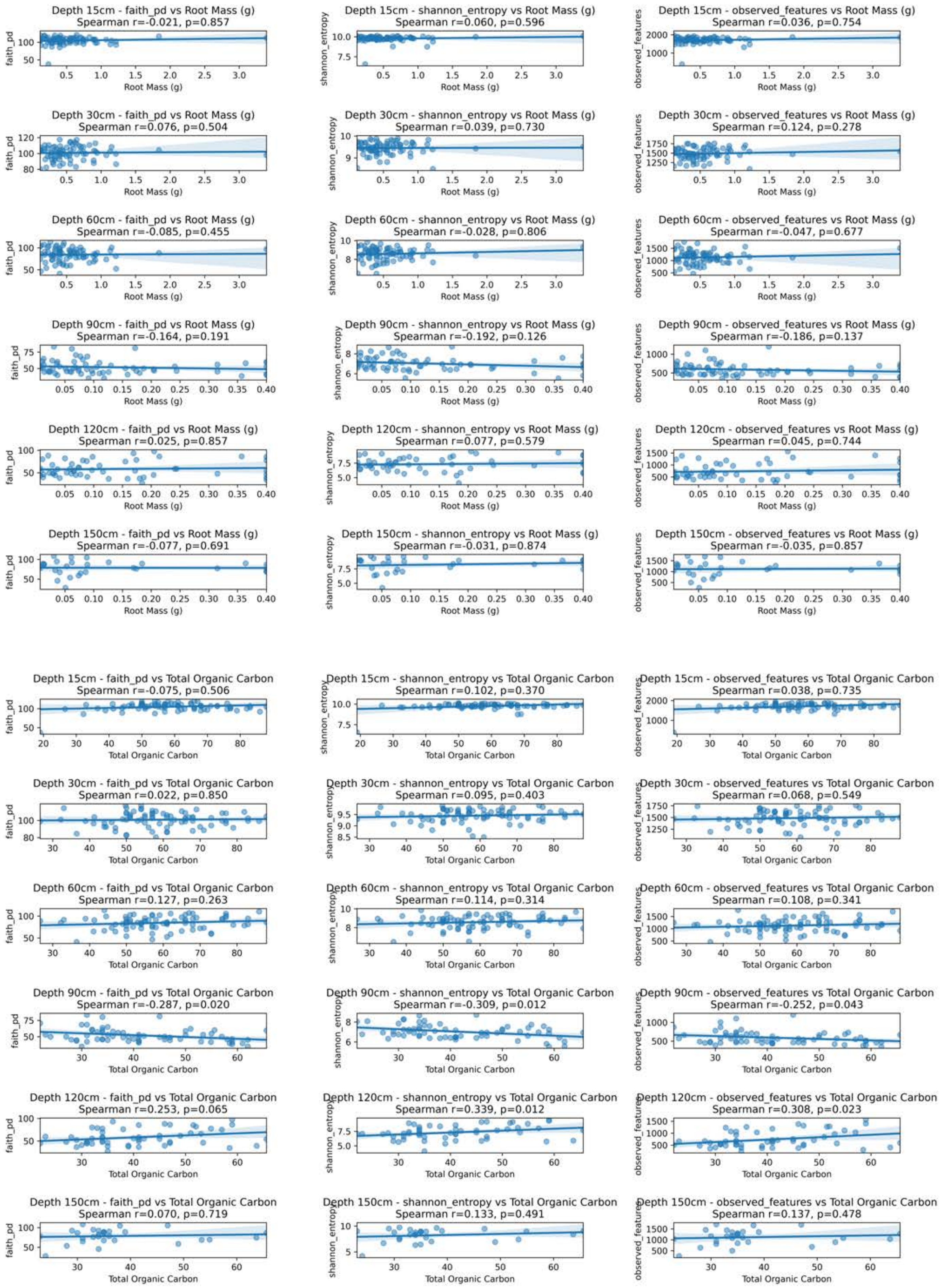

Fig S2: Changes in alpha diversity levels with root mass and total organic carbon at each depth displaying Spearman's rank correlation coefficients and p-values in the plot titles.

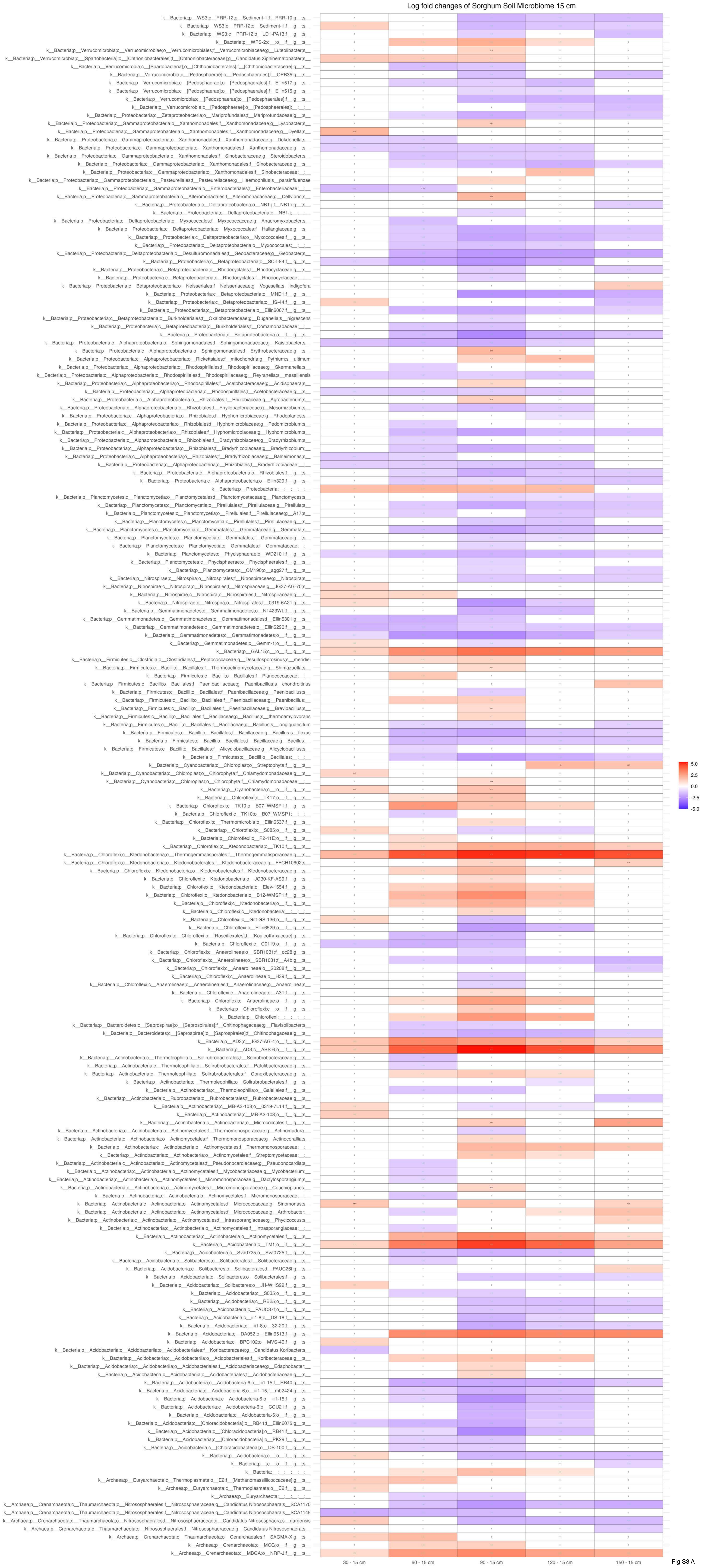

Log fold changes of Sorghum Soil Microbiome 15 cm
30 - 15 cm
60 - 15 cm
90 - 15 cm
120 - 15 cm
150 - 15 cm
5.0
2.5
0.0
-2.5
-5.0

Fig S3 A

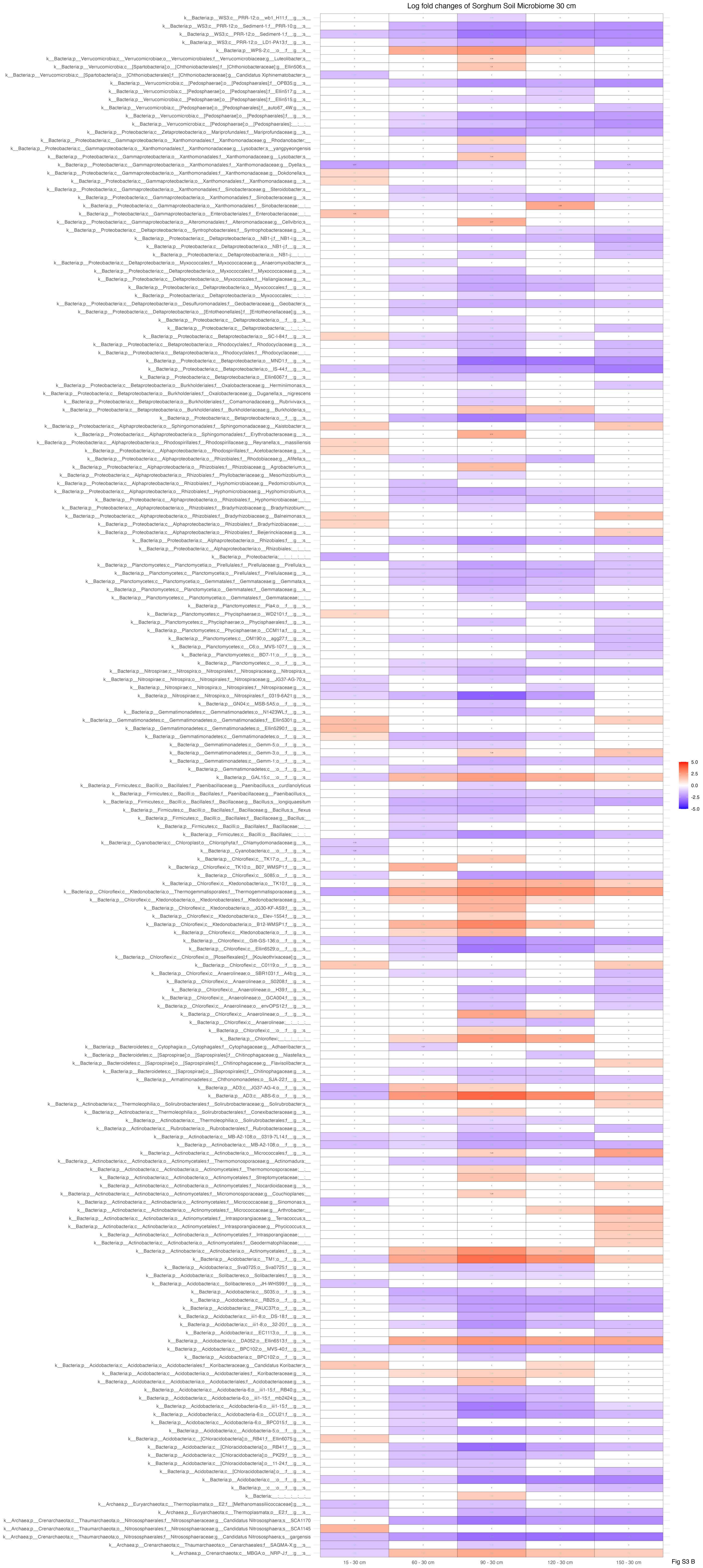
Log fold changes of Sorghum Soil Microbiome 30 cm
5.0
2.5
0.0
-2.5
-5.0
15 - 30 cm
60 - 30 cm
90 - 30 cm
120 - 30 cm
150 - 30 cm

Fig S3 B

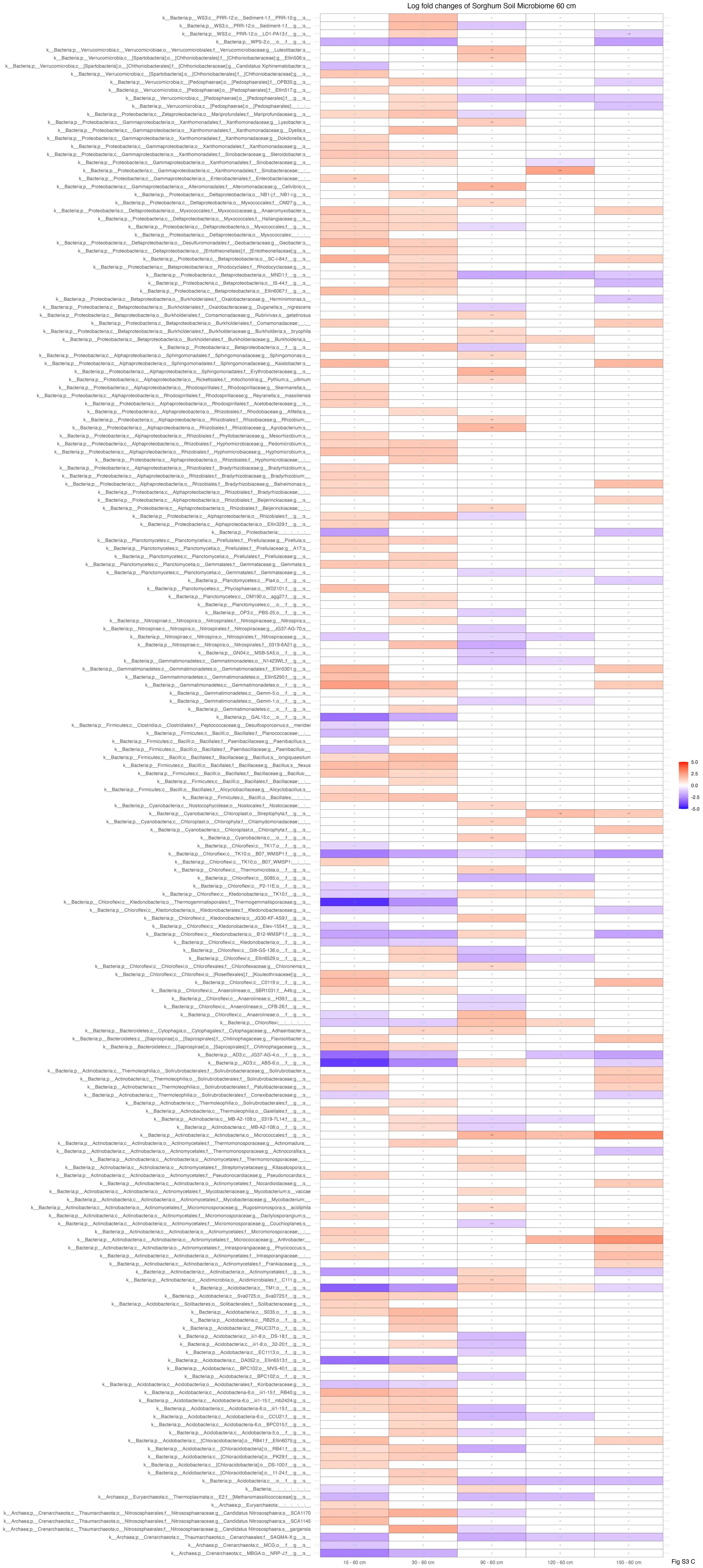
Log fold changes of Sorghum Soil Microbiome 60 cm
15 - 60 cm
30 - 60 cm
90 - 60 cm
120 - 60 cm
150 - 60 cm
5.0
2.5
0.0
-2.5
-5.0

Fig S3 C

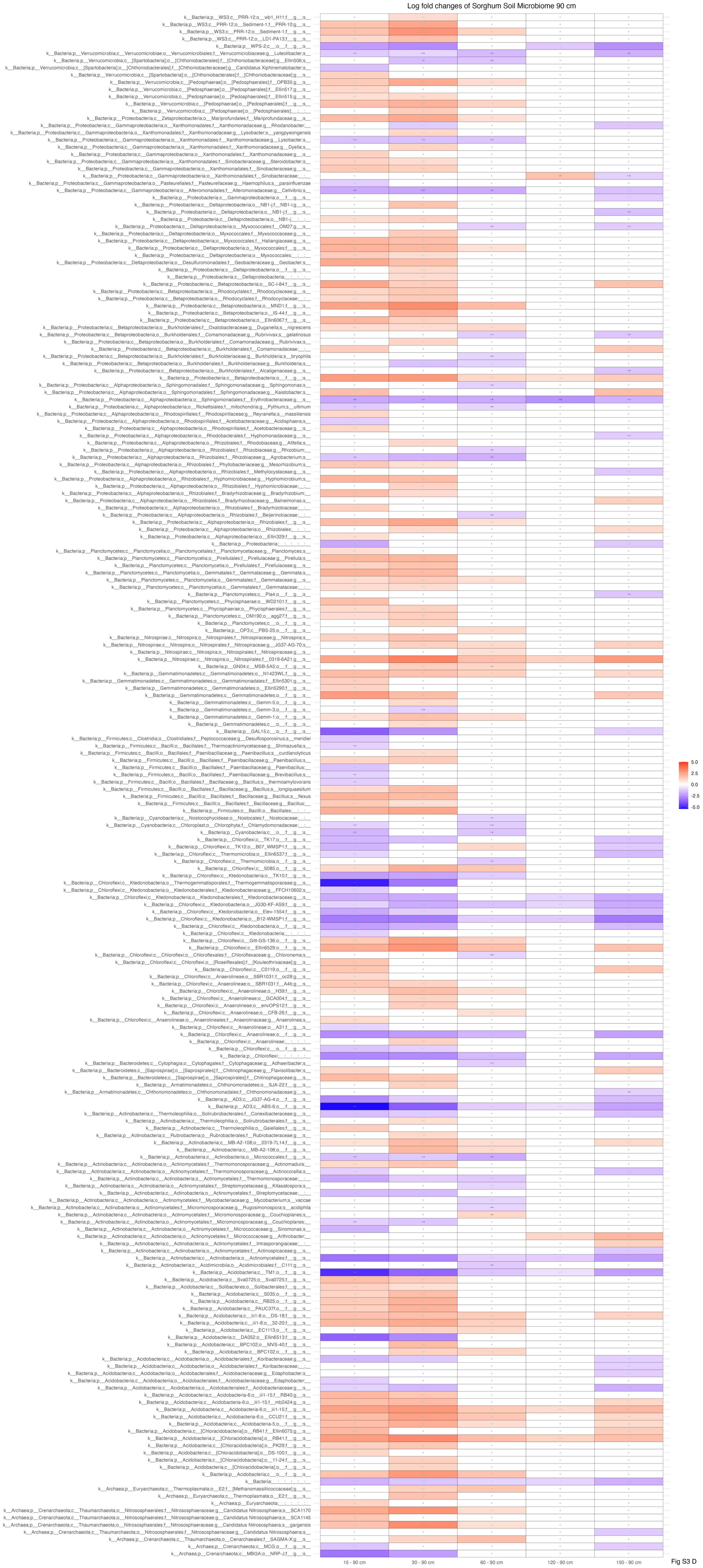
Log fold changes of Sorghum Soil Microbiome 90 cm
15 - 90 cm
30 - 90 cm
60 - 90 cm
120 - 90 cm
150 - 90 cm
5.0
2.5
0.0
-2.5
-5.0

Fig S3 D

Log fold changes of Sorghum Soil Microbiome 120 cm

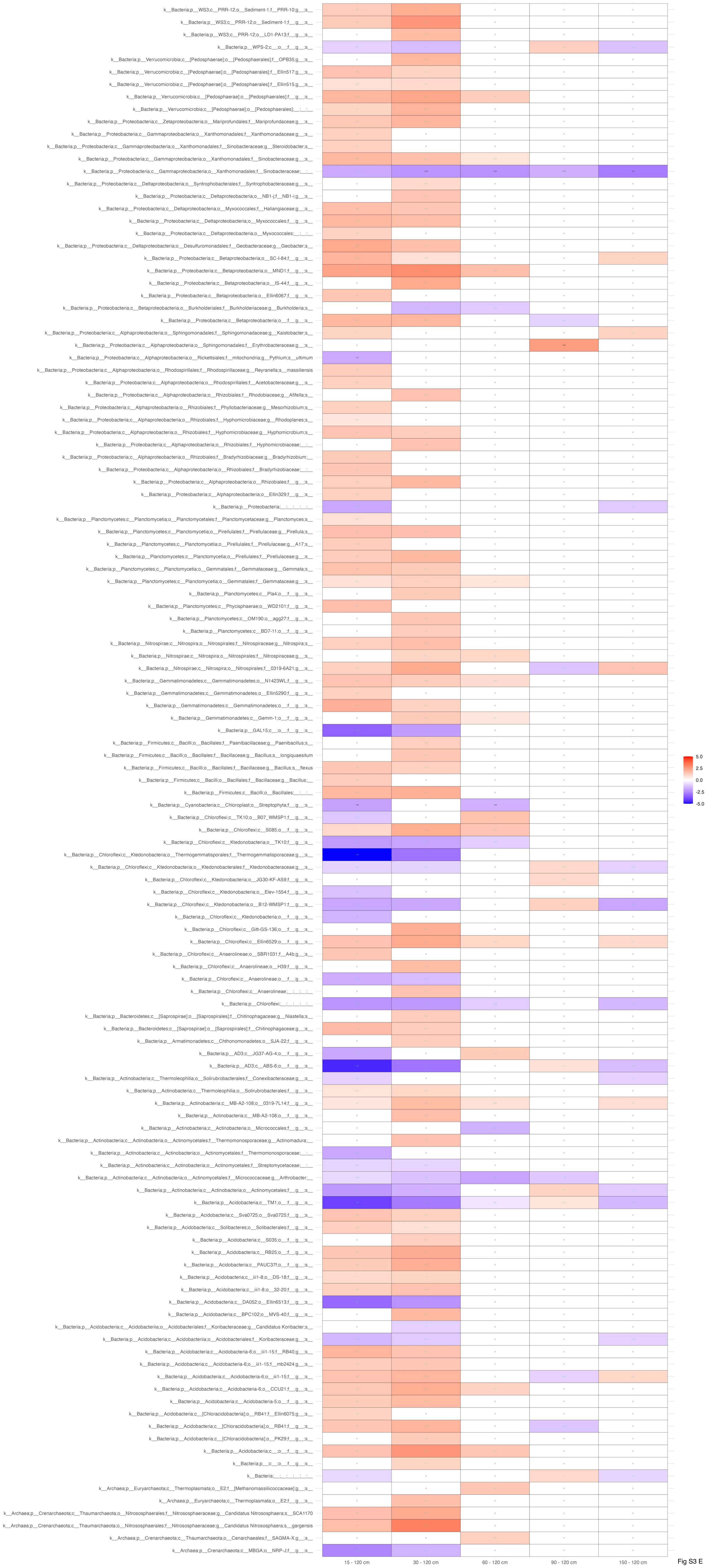

Fig S3 E

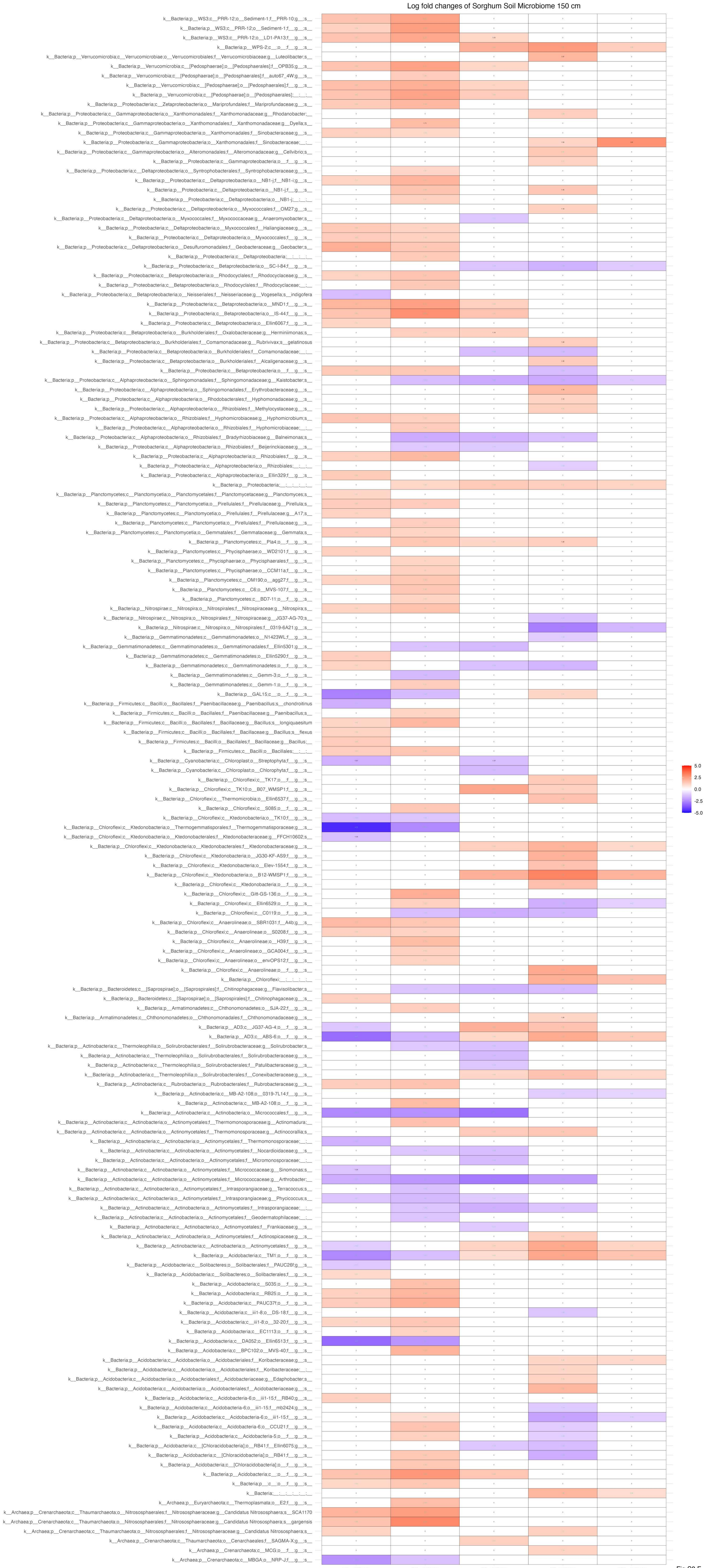
Log fold changes of Sorghum Soil Microbiome 150 cm
k__Bacteria;p__WS3;c__PRR-12;o__Sediment-1;f__PRR-10;g__;s__
k__Bacteria;p__WS3;c__PRR-12;o__Sediment-1;f__;g__;s__
k__Bacteria;p__WS3;c__PRR-12;o__LD1-PA13;f__;g__;s__
k__Bacteria;p__WPS-2;c__;o__;f__;g__;s__
k__Bacteria;p__Verrucomicrobia;c__Verrucomicrobiae;o__Verrucomicrobiales;f__Verrucomicrobiaceae;g__Luteolibacter;s__
k__Bacteria;p__Verrucomicrobia;c__[Pedosphaerae];o__[Pedosphaerales];f__OPB35;g__;s__
k__Bacteria;p__Verrucomicrobia;c__[Pedosphaerae];o__[Pedosphaerales];f__auto67_4W;g__;s__
k__Bacteria;p__Verrucomicrobia;c__[Pedosphaerae];o__[Pedosphaerales];f__;g__;s__
k__Bacteria;p__Verrucomicrobia;c__[Pedosphaerae];o__[Pedosphaerales];__;__;__
k__Bacteria;p__Proteobacteria;c__Zetaproteobacteria;o__Mariprofundales;f__Mariprofundaceae;g__;s__
k__Bacteria;p__Proteobacteria;c__Gammaproteobacteria;o__Xanthomonadales;f__Xanthomonadaceae;g__Rhodanobacter;__
k__Bacteria;p__Proteobacteria;c__Gammaproteobacteria;o__Xanthomonadales;f__Xanthomonadaceae;g__Dyella;s__
k__Bacteria;p__Proteobacteria;c__Gammaproteobacteria;o__Xanthomonadales;f__Sinobacteraceae;g__;s__
k__Bacteria;p__Proteobacteria;c__Gammaproteobacteria;o__Xanthomonadales;f__Sinobacteraceae;__;__
k__Bacteria;p__Proteobacteria;c__Gammaproteobacteria;o__Alteromonadales;f__Alteromonadaceae;g__Cellvibrio;s__
k__Bacteria;p__Proteobacteria;c__Gammaproteobacteria;o__;f__;g__;s__
k__Bacteria;p__Proteobacteria;c__Deltaproteobacteria;o__Syntrophobacterales;f__Syntrophobacteraceae;g__;s__
k__Bacteria;p__Proteobacteria;c__Deltaproteobacteria;o__NB1-j;f__NB1-i;g__;s__
k__Bacteria;p__Proteobacteria;c__Deltaproteobacteria;o__NB1-j;f__;g__;s__
k__Bacteria;p__Proteobacteria;c__Deltaproteobacteria;o__NB1-j;__;__;__
k__Bacteria;p__Proteobacteria;c__Deltaproteobacteria;o__Myxococcales;f__OM27;g__;s__
k__Bacteria;p__Proteobacteria;c__Deltaproteobacteria;o__Myxococcales;f__Myxococcaceae;g__Anaeromyxobacter;s__
k__Bacteria;p__Proteobacteria;c__Deltaproteobacteria;o__Myxococcales;f__Haliangiaceae;g__;s__
k__Bacteria;p__Proteobacteria;c__Deltaproteobacteria;o__Myxococcales;f__;g__;s__
k__Bacteria;p__Proteobacteria;c__Deltaproteobacteria;o__Desulfuromonadales;f__Geobacteraceae;g__Geobacter;s__
k__Bacteria;p__Proteobacteria;c__Deltaproteobacteria;__;__;__;__
k__Bacteria;p__Proteobacteria;c__Betaproteobacteria;o__SC-I-84;f__;g__;s__
k__Bacteria;p__Proteobacteria;c__Betaproteobacteria;o__Rhodocyclales;f__Rhodocyclaceae;g__;s__
k__Bacteria;p__Proteobacteria;c__Betaproteobacteria;o__Rhodocyclales;f__Rhodocyclaceae;__;__
k__Bacteria;p__Proteobacteria;c__Betaproteobacteria;o__Neisseriales;f__Neisseriaceae;g__Vogesella;s__indigofera
k__Bacteria;p__Proteobacteria;c__Betaproteobacteria;o__MND1;f__;g__;s__
k__Bacteria;p__Proteobacteria;c__Betaproteobacteria;o__IS-44;f__;g__;s__
k__Bacteria;p__Proteobacteria;c__Betaproteobacteria;o__Ellin6067;f__;g__;s__
k__Bacteria;p__Proteobacteria;c__Betaproteobacteria;o__Burkholderiales;f__Oxalobacteraceae;g__Herminiimonas;s__
k__Bacteria;p__Proteobacteria;c__Betaproteobacteria;o__Burkholderiales;f__Comamonadaceae;g__Rubrivivax;s__gelatinosus
k__Bacteria;p__Proteobacteria;c__Betaproteobacteria;o__Burkholderiales;f__Comamonadaceae;__;__
k__Bacteria;p__Proteobacteria;c__Betaproteobacteria;o__Burkholderiales;f__Alcaligenaceae;g__;s__
k__Bacteria;p__Proteobacteria;c__Betaproteobacteria;o__;f__;g__;s__
k__Bacteria;p__Proteobacteria;c__Alphaproteobacteria;o__Sphingomonadales;f__Sphingomonadaceae;g__Kaistobacter;s__
k__Bacteria;p__Proteobacteria;c__Alphaproteobacteria;o__Sphingomonadales;f__Erythrobacteraceae;g__;s__
k__Bacteria;p__Proteobacteria;c__Alphaproteobacteria;o__Rhodobacterales;f__Hyphomonadaceae;g__;s__
k__Bacteria;p__Proteobacteria;c__Alphaproteobacteria;o__Rhizobiales;f__Methylocystaceae;g__;s__
k__Bacteria;p__Proteobacteria;c__Alphaproteobacteria;o__Rhizobiales;f__Hyphomicrobiaceae;g__Hyphomicrobium;s__
k__Bacteria;p__Proteobacteria;c__Alphaproteobacteria;o__Rhizobiales;f__Hyphomicrobiaceae;__;__
k__Bacteria;p__Proteobacteria;c__Alphaproteobacteria;o__Rhizobiales;f__Bradyrhizobiaceae;g__Balneimonas;s__
k__Bacteria;p__Proteobacteria;c__Alphaproteobacteria;o__Rhizobiales;f__Beijerinckiaceae;g__;s__
k__Bacteria;p__Proteobacteria;c__Alphaproteobacteria;o__Rhizobiales;f__;g__;s__
k__Bacteria;p__Proteobacteria;c__Alphaproteobacteria;o__Rhizobiales;__;__;__
k__Bacteria;p__Proteobacteria;c__Alphaproteobacteria;o__Ellin329;f__;g__;s__
k__Bacteria;p__Proteobacteria;__;__;__;__;__
k__Bacteria;p__Planctomycetes;c__Planctomycetia;o__Planctomycetales;f__Planctomycetaceae;g__Planctomyces;s__
k__Bacteria;p__Planctomycetes;c__Planctomycetia;o__Pirellulales;f__Pirellulaceae;g__Pirellula;s__
k__Bacteria;p__Planctomycetes;c__Planctomycetia;o__Pirellulales;f__Pirellulaceae;g__A17;s__
k__Bacteria;p__Planctomycetes;c__Planctomycetia;o__Pirellulales;f__Pirellulaceae;g__;s__
k__Bacteria;p__Planctomycetes;c__Planctomycetia;o__Gemmatales;f__Gemmataceae;g__Gemmata;s__
k__Bacteria;p__Planctomycetes;c__Pla4;o__;f__;g__;s__
k__Bacteria;p__Planctomycetes;c__Phycisphaerae;o__WD2101;f__;g__;s__
k__Bacteria;p__Planctomycetes;c__Phycisphaerae;o__Phycisphaerales;f__;g__;s__
k__Bacteria;p__Planctomycetes;c__Phycisphaerae;o__CCM11a;f__;g__;s__
k__Bacteria;p__Planctomycetes;c__OM190;o__agg27;f__;g__;s__
k__Bacteria;p__Planctomycetes;c__C6;o__MVS-107;f__;g__;s__
k__Bacteria;p__Planctomycetes;c__BD7-11;o__;f__;g__;s__
k__Bacteria;p__Nitrospirae;c__Nitrospira;o__Nitrospirales;f__Nitrospiraceae;g__Nitrospira;s__
k__Bacteria;p__Nitrospirae;c__Nitrospira;o__Nitrospirales;f__Nitrospiraceae;g__JG37-AG-70;s__
k__Bacteria;p__Nitrospirae;c__Nitrospira;o__Nitrospirales;f__0319-6A21;g__;s__
k__Bacteria;p__Gemmatimonadetes;c__Gemmatimonadetes;o__N1423WL;f__;g__;s__
k__Bacteria;p__Gemmatimonadetes;c__Gemmatimonadetes;o__Gemmatimonadales;f__Ellin5301;g__;s__
k__Bacteria;p__Gemmatimonadetes;c__Gemmatimonadetes;o__Ellin5290;f__;g__;s__
k__Bacteria;p__Gemmatimonadetes;c__Gemmatimonadetes;o__;f__;g__;s__
k__Bacteria;p__Gemmatimonadetes;c__Gemm-3;o__;f__;g__;s__
k__Bacteria;p__Gemmatimonadetes;c__Gemm-1;o__;f__;g__;s__
k__Bacteria;p__GAL15;c__;o__;f__;g__;s__
k__Bacteria;p__Firmicutes;c__Bacilli;o__Bacillales;f__Paenibacillaceae;g__Paenibacillus;s__chondroitinus
k__Bacteria;p__Firmicutes;c__Bacilli;o__Bacillales;f__Paenibacillaceae;g__Paenibacillus;s__
k__Bacteria;p__Firmicutes;c__Bacilli;o__Bacillales;f__Bacillaceae;g__Bacillus;s__longiquaesitum
k__Bacteria;p__Firmicutes;c__Bacilli;o__Bacillales;f__Bacillaceae;g__Bacillus;s__flexus
k__Bacteria;p__Firmicutes;c__Bacilli;o__Bacillales;f__Bacillaceae;g__Bacillus;__
k__Bacteria;p__Firmicutes;c__Bacilli;o__Bacillales;__;__;__
k__Bacteria;p__Cyanobacteria;c__Chloroplast;o__Streptophyta;f__;g__;s__
k__Bacteria;p__Cyanobacteria;c__Chloroplast;o__Chlorophyta;f__;g__;s__
k__Bacteria;p__Chloroflexi;c__TK17;o__;f__;g__;s__
k__Bacteria;p__Chloroflexi;c__TK10;o__B07_WMSP1;f__;g__;s__
k__Bacteria;p__Chloroflexi;c__Thermomicrobia;o__Ellin6537;f__;g__;s__
k__Bacteria;p__Chloroflexi;c__S085;o__;f__;g__;s__
k__Bacteria;p__Chloroflexi;c__Ktedonobacteria;o__TK10;f__;g__;s__
k__Bacteria;p__Chloroflexi;c__Ktedonobacteria;o__Thermogemmatisporales;f__Thermogemmatisporaceae;g__;s__
k__Bacteria;p__Chloroflexi;c__Ktedonobacteria;o__Ktedonobacterales;f__Ktedonobacteraceae;g__FFCH10602;s__
k__Bacteria;p__Chloroflexi;c__Ktedonobacteria;o__Ktedonobacterales;f__Ktedonobacteraceae;g__;s__
k__Bacteria;p__Chloroflexi;c__Ktedonobacteria;o__JG30-KF-AS9;f__;g__;s__
k__Bacteria;p__Chloroflexi;c__Ktedonobacteria;o__Elev-1554;f__;g__;s__
k__Bacteria;p__Chloroflexi;c__Ktedonobacteria;o__B12-WMSP1;f__;g__;s__
k__Bacteria;p__Chloroflexi;c__Ktedonobacteria;o__;f__;g__;s__
k__Bacteria;p__Chloroflexi;c__Gitt-GS-136;o__;f__;g__;s__
k__Bacteria;p__Chloroflexi;c__Ellin6529;o__;f__;g__;s__
k__Bacteria;p__Chloroflexi;c__C0119;o__;f__;g__;s__
k__Bacteria;p__Chloroflexi;c__Anaerolineae;o__SBR1031;f__A4b;g__;s__
k__Bacteria;p__Chloroflexi;c__Anaerolineae;o__S0208;f__;g__;s__
k__Bacteria;p__Chloroflexi;c__Anaerolineae;o__H39;f__;g__;s__
k__Bacteria;p__Chloroflexi;c__Anaerolineae;o__GCA004;f__;g__;s__
k__Bacteria;p__Chloroflexi;c__Anaerolineae;o__envOPS12;f__;g__;s__
k__Bacteria;p__Chloroflexi;c__Anaerolineae;o__;f__;g__;s__
k__Bacteria;p__Chloroflexi;__;__;__;__;__
k__Bacteria;p__Bacteroidetes;c__[Saprospirae];o__[Saprospirales];f__Chitinophagaceae;g__Flavisolibacter;s__
k__Bacteria;p__Bacteroidetes;c__[Saprospirae];o__[Saprospirales];f__Chitinophagaceae;g__;s__
k__Bacteria;p__Armatimonadetes;c__Chthonomonadetes;o__SJA-22;f__;g__;s__
k__Bacteria;p__Armatimonadetes;c__Chthonomonadetes;o__Chthonomonadales;f__Chthonomonadaceae;g__;s__
k__Bacteria;p__AD3;c__JG37-AG-4;o__;f__;g__;s__
k__Bacteria;p__AD3;c__ABS-6;o__;f__;g__;s__
k__Bacteria;p__Actinobacteria;c__Thermoleophilia;o__Solirubrobacterales;f__Solirubrobacteraceae;g__Solirubrobacter;s__
k__Bacteria;p__Actinobacteria;c__Thermoleophilia;o__Solirubrobacterales;f__Solirubrobacteraceae;g__;s__
k__Bacteria;p__Actinobacteria;c__Thermoleophilia;o__Solirubrobacterales;f__Patulibacteraceae;g__;s__
k__Bacteria;p__Actinobacteria;c__Thermoleophilia;o__Solirubrobacterales;f__Conexibacteraceae;g__;s__
k__Bacteria;p__Actinobacteria;c__Rubrobacteria;o__Rubrobacterales;f__Rubrobacteraceae;g__;s__
k__Bacteria;p__Actinobacteria;c__MB-A2-108;o__0319-7L14;f__;g__;s__
k__Bacteria;p__Actinobacteria;c__MB-A2-108;o__;f__;g__;s__
k__Bacteria;p__Actinobacteria;c__Actinobacteria;o__Micrococcales;f__;g__;s__
k__Bacteria;p__Actinobacteria;c__Actinobacteria;o__Actinomycetales;f__Thermomonosporaceae;g__Actinomadura;__
k__Bacteria;p__Actinobacteria;c__Actinobacteria;o__Actinomycetales;f__Thermomonosporaceae;g__Actinocorallia;s__
k__Bacteria;p__Actinobacteria;c__Actinobacteria;o__Actinomycetales;f__Thermomonosporaceae;__;__
k__Bacteria;p__Actinobacteria;c__Actinobacteria;o__Actinomycetales;f__Nocardioidaceae;g__;s__
k__Bacteria;p__Actinobacteria;c__Actinobacteria;o__Actinomycetales;f__Micromonosporaceae;__;__
k__Bacteria;p__Actinobacteria;c__Actinobacteria;o__Actinomycetales;f__Micrococcaceae;g__Sinomonas;s__
k__Bacteria;p__Actinobacteria;c__Actinobacteria;o__Actinomycetales;f__Micrococcaceae;g__Arthrobacter;__
k__Bacteria;p__Actinobacteria;c__Actinobacteria;o__Actinomycetales;f__Intrasporangiaceae;g__Terracoccus;s__
k__Bacteria;p__Actinobacteria;c__Actinobacteria;o__Actinomycetales;f__Intrasporangiaceae;g__Phycicoccus;s__
k__Bacteria;p__Actinobacteria;c__Actinobacteria;o__Actinomycetales;f__Intrasporangiaceae;__;__
k__Bacteria;p__Actinobacteria;c__Actinobacteria;o__Actinomycetales;f__Geodermatophilaceae;__;__
k__Bacteria;p__Actinobacteria;c__Actinobacteria;o__Actinomycetales;f__Frankiaceae;g__;s__
k__Bacteria;p__Actinobacteria;c__Actinobacteria;o__Actinomycetales;f__Actinospicaceae;g__;s__
k__Bacteria;p__Actinobacteria;c__Actinobacteria;o__Actinomycetales;f__;g__;s__
k__Bacteria;p__Acidobacteria;c__TM1;o__;f__;g__;s__
k__Bacteria;p__Acidobacteria;c__Solibacteres;o__Solibacterales;f__PAUC26f;g__;s__
k__Bacteria;p__Acidobacteria;c__Solibacteres;o__Solibacterales;f__;g__;s__
k__Bacteria;p__Acidobacteria;c__S035;o__;f__;g__;s__
k__Bacteria;p__Acidobacteria;c__RB25;o__;f__;g__;s__
k__Bacteria;p__Acidobacteria;c__PAUC37f;o__;f__;g__;s__
k__Bacteria;p__Acidobacteria;c__iii1-8;o__DS-18;f__;g__;s__
k__Bacteria;p__Acidobacteria;c__iii1-8;o__32-20;f__;g__;s__
k__Bacteria;p__Acidobacteria;c__EC1113;o__;f__;g__;s__
k__Bacteria;p__Acidobacteria;c__DA052;o__Ellin6513;f__;g__;s__
k__Bacteria;p__Acidobacteria;c__BPC102;o__MVS-40;f__;g__;s__
k__Bacteria;p__Acidobacteria;c__Acidobacteriia;o__Acidobacteriales;f__Koribacteraceae;g__;s__
k__Bacteria;p__Acidobacteria;c__Acidobacteriia;o__Acidobacteriales;f__Koribacteraceae;__;__
k__Bacteria;p__Acidobacteria;c__Acidobacteriia;o__Acidobacteriales;f__Acidobacteriaceae;g__Edaphobacter;s__
k__Bacteria;p__Acidobacteria;c__Acidobacteriia;o__Acidobacteriales;f__Acidobacteriaceae;g__;s__
k__Bacteria;p__Acidobacteria;c__Acidobacteria-6;o__iii1-15;f__RB40;g__;s__
k__Bacteria;p__Acidobacteria;c__Acidobacteria-6;o__iii1-15;f__mb2424;g__;s__
k__Bacteria;p__Acidobacteria;c__Acidobacteria-6;o__iii1-15;f__;g__;s__
k__Bacteria;p__Acidobacteria;c__Acidobacteria-6;o__CCU21;f__;g__;s__
k__Bacteria;p__Acidobacteria;c__Acidobacteria-5;o__;f__;g__;s__
k__Bacteria;p__Acidobacteria;c__[Chloracidobacteria];o__RB41;f__Ellin6075;g__;s__
k__Bacteria;p__Acidobacteria;c__[Chloracidobacteria];o__RB41;f__;g__;s__
k__Bacteria;p__Acidobacteria;c__[Chloracidobacteria];o__;f__;g__;s__
k__Bacteria;p__Acidobacteria;c__;o__;f__;g__;s__
k__Bacteria;p__;c__;o__;f__;g__;s__
k__Bacteria;__;__;__;__;__;__
k__Archaea;p__Euryarchaeota;c__Thermoplasmata;o__E2;f__;g__;s__
k__Archaea;p__Crenarchaeota;c__Thaumarchaeota;o__Nitrososphaerales;f__Nitrososphaeraceae;g__Candidatus Nitrososphaera;s__SCA1170
k__Archaea;p__Crenarchaeota;c__Thaumarchaeota;o__Nitrososphaerales;f__Nitrososphaeraceae;g__Candidatus Nitrososphaera;s__gargensis
k__Archaea;p__Crenarchaeota;c__Thaumarchaeota;o__Nitrososphaerales;f__Nitrososphaeraceae;g__Candidatus Nitrososphaera;s__
k__Archaea;p__Crenarchaeota;c__Thaumarchaeota;o__Cenarchaeales;f__SAGMA-X;g__;s__
k__Archaea;p__Crenarchaeota;c__MCG;o__;f__;g__;s__
k__Archaea;p__Crenarchaeota;c__MBGA;o__NRP-J;f__;g__;s__
15 - 150 cm
30 - 150 cm
60 - 150 cm
90 - 150 cm
120 - 150 cm
5.0
2.5
0.0
-2.5
-5.0

Fig S3 F

Fig S3: Log fold changes of microbial species at various depths. A-F*)* Heatmaps displaying all of the log fold changes (LFC) of the *S. bicolor* soil microbiome across at different depths compared to the reference depth A) 15 cm, B) 30 cm, C) 60 cm, D) 90 cm, E) 120 cm and F) 150 cm. The y-axes display the taxa arranged in alphabetical order. The x-axes show the different soil depth comparisons. The color gradient ranges from blue, indicating negative log fold changes, to red, indicating positive log fold changes, with white representing no change. The midpoint of the scale is set to 0, with limits ranging from -5.4 to 5.4. Comparisons that have successfully passed the sensitivity analysis for pseudo-count addition are denoted by aquamarine text. Taxa with no significant values in any comparisons were excluded to focus on the relevant data.
